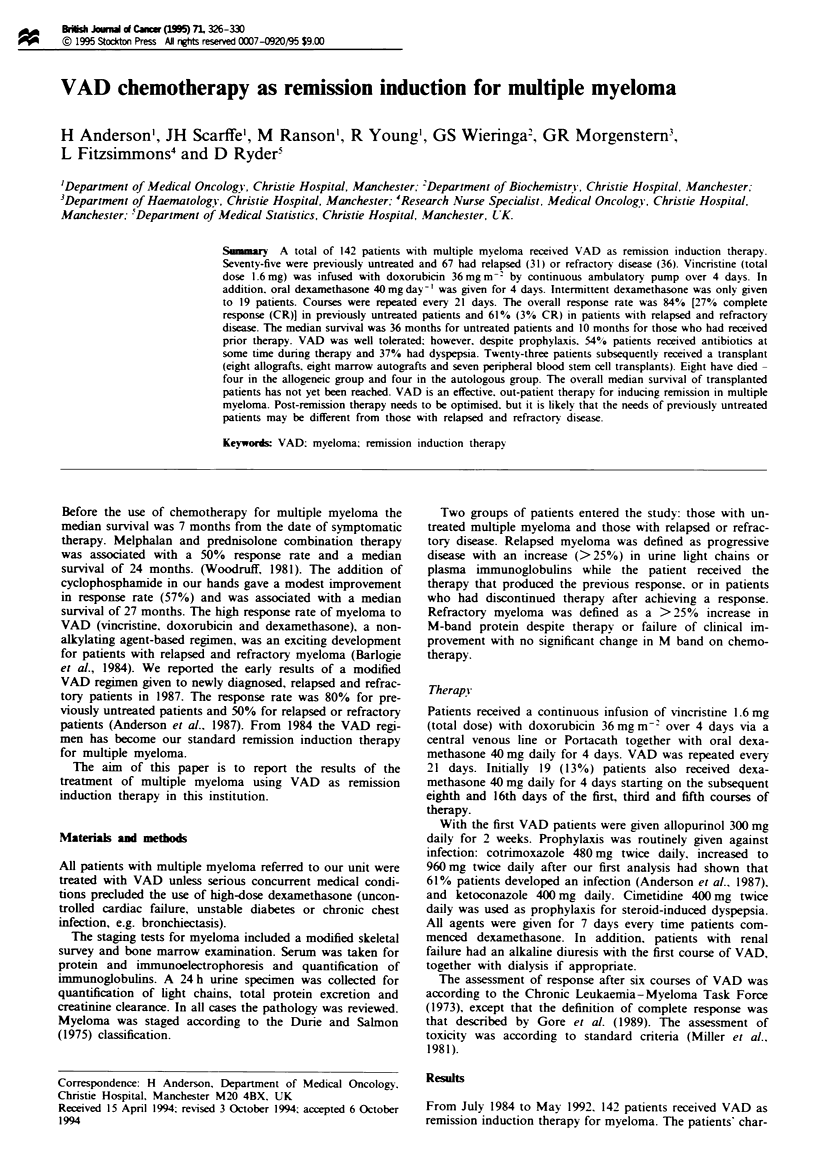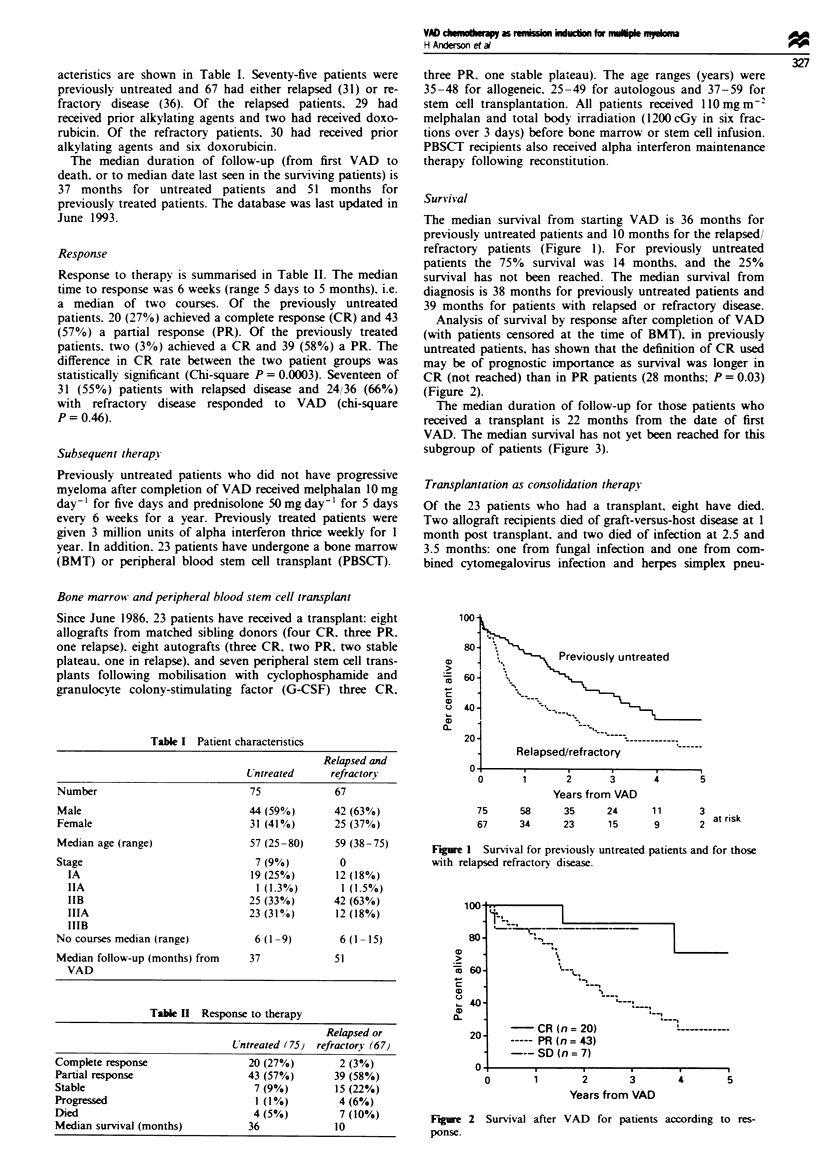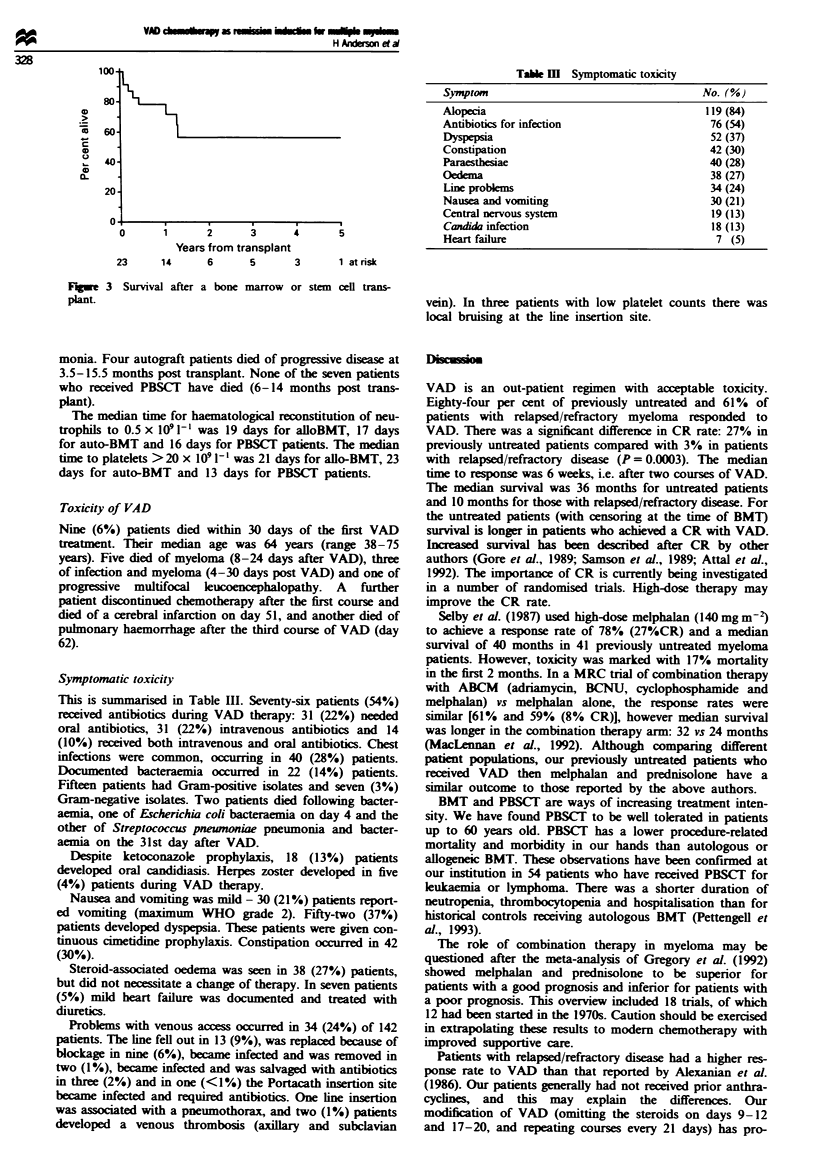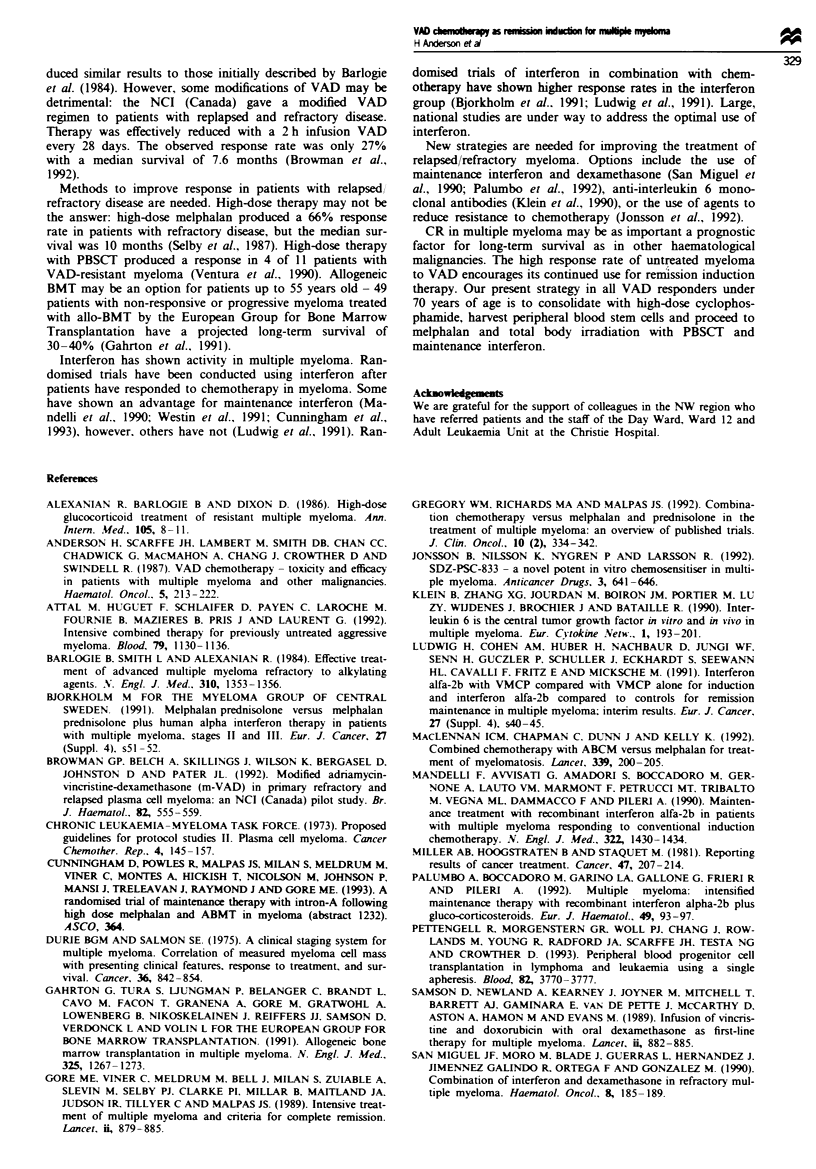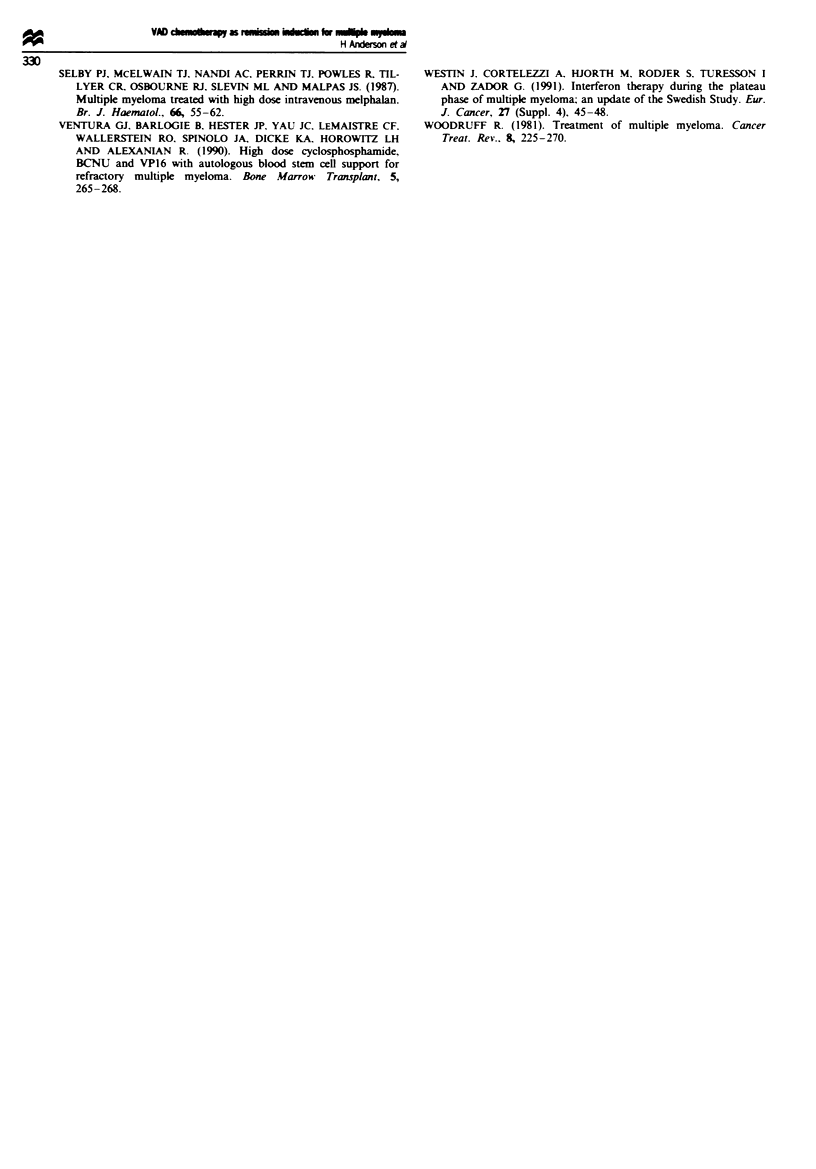# VAD chemotherapy as remission induction for multiple myeloma.

**DOI:** 10.1038/bjc.1995.65

**Published:** 1995-02

**Authors:** H. Anderson, J. H. Scarffe, M. Ranson, R. Young, G. S. Wieringa, G. R. Morgenstern, L. Fitzsimmons, D. Ryder

**Affiliations:** Department of Medical Oncology, Christie Hospital, Manchester, UK.

## Abstract

A total of 142 patients with multiple myeloma received VAD as remission induction therapy. Seventy-five were previously untreated and 67 had relapsed (31) or refractory disease (36). Vincristine (total dose 1.6 mg) was infused with doxorubicin 36 mg m-2 by continuous ambulatory pump over 4 days. In addition, oral dexamethasone 40 mg day-1 was given for 4 days. Intermittent dexamethasone was only given to 19 patients. Courses were repeated every 21 days. The overall response rate was 84% [27% complete response (CR)] in previously untreated patients and 61% (3% CR) in patients with relapsed and refractory disease. The median survival was 36 months for untreated patients and 10 months for those who had received prior therapy. VAD was well tolerated; however, despite prophylaxis, 54% patients received antibiotics at some time during therapy and 37% had dyspepsia. Twenty-three patients subsequently received a transplant (eight allografts, eight marrow autografts and seven peripheral blood stem cell transplants). Eight have died-four in the allogeneic group and four in the autologous group. The overall median survival of transplanted patients has not yet been reached. VAD is an effective, out-patient therapy for inducing remission in multiple myeloma. Post-remission therapy needs to be optimised, but it is likely that the needs of previously untreated patients may be different from those with relapsed and refractory disease.


British Journal of Cancer (1995) 71, 326-330

© 1995 Stockton Press All rights reserved 0007-0920/95 $9.00

### VAD chemotherapy as remission induction for multiple myeloma

H Anderson[1], JH Scarffe[1], M Ranson[1], R Young[1], GS Wieringa[2], GR Morgenstern[3], L Fitzsimmons[4] and D Ryder[5]

[1]*Department of Medical Oncology, Christie Hospital, Manchester;* [2]*Department of Biochemistry, Christie Hospital, Manchester;* [3]*Department of Haematology, Christie Hospital, Manchester;* [4]*Research Nurse Specialist, Medical Oncology, Christie Hospital, Manchester;* [5]*Department of Medical Statistics, Christie Hospital, Manchester, UK.*

**Summary** A total of 142 patients with multiple myeloma received VAD as remission induction therapy. Seventy-five were previously untreated and 67 had relapsed (31) or refractory disease (36). Vincristine (total dose 1.6 mg) was infused with doxorubicin 36 mg m$^{-2}$ by continuous ambulatory pump over 4 days. In addition, oral dexamethasone 40 mg day$^{-1}$ was given for 4 days. Intermittent dexamethasone was only given to 19 patients. Courses were repeated every 21 days. The overall response rate was 84% [27% complete response (CR)] in previously untreated patients and 61% (3% CR) in patients with relapsed and refractory disease. The median survival was 36 months for untreated patients and 10 months for those who had received prior therapy. VAD was well tolerated; however, despite prophylaxis, 54% patients received antibiotics at some time during therapy and 37% had dyspepsia. Twenty-three patients subsequently received a transplant (eight allografts, eight marrow autografts and seven peripheral blood stem cell transplants). Eight have died - four in the allogeneic group and four in the autologous group. The overall median survival of transplanted patients has not yet been reached. VAD is an effective, out-patient therapy for inducing remission in multiple myeloma. Post-remission therapy needs to be optimised, but it is likely that the needs of previously untreated patients may be different from those with relapsed and refractory disease.

**Keywords:** VAD; myeloma; remission induction therapy

Before the use of chemotherapy for multiple myeloma the median survival was 7 months from the date of symptomatic therapy. Melphalan and prednisolone combination therapy was associated with a 50% response rate and a median survival of 24 months. (Woodruff, 1981). The addition of cyclophosphamide in our hands gave a modest improvement in response rate (57%) and was associated with a median survival of 27 months. The high response rate of myeloma to VAD (vincristine, doxorubicin and dexamethasone), a non-alkylating agent-based regimen, was an exciting development for patients with relapsed and refractory myeloma (Barlogie *et al.*, 1984). We reported the early results of a modified VAD regimen given to newly diagnosed, relapsed and refractory patients in 1987. The response rate was 80% for previously untreated patients and 50% for relapsed or refractory patients (Anderson *et al.*, 1987). From 1984 the VAD regimen has become our standard remission induction therapy for multiple myeloma.

The aim of this paper is to report the results of the treatment of multiple myeloma using VAD as remission induction therapy in this institution.

### Materials and methods

All patients with multiple myeloma referred to our unit were treated with VAD unless serious concurrent medical conditions precluded the use of high-dose dexamethasone (uncontrolled cardiac failure, unstable diabetes or chronic chest infection, e.g. bronchiectasis).

The staging tests for myeloma included a modified skeletal survey and bone marrow examination. Serum was taken for protein and immunoelectrophoresis and quantification of immunoglobulins. A 24 h urine specimen was collected for quantification of light chains, total protein excretion and creatinine clearance. In all cases the pathology was reviewed. Myeloma was staged according to the Durie and Salmon (1975) classification.

Correspondence: H Anderson, Department of Medical Oncology, Christie Hospital, Manchester M20 4BX, UK
Received 15 April 1994; revised 3 October 1994; accepted 6 October 1994

Two groups of patients entered the study: those with untreated multiple myeloma and those with relapsed or refractory disease. Relapsed myeloma was defined as progressive disease with an increase (>25%) in urine light chains or plasma immunoglobulins while the patient received the therapy that produced the previous response, or in patients who had discontinued therapy after achieving a response. Refractory myeloma was defined as a >25% increase in M-band protein despite therapy or failure of clinical improvement with no significant change in M band on chemotherapy.

### *Therapy*

Patients received a continuous infusion of vincristine 1.6 mg (total dose) with doxorubicin 36 mg m$^{-2}$ over 4 days via a central venous line or Portacath together with oral dexamethasone 40 mg daily for 4 days. VAD was repeated every 21 days. Initially 19 (13%) patients also received dexamethasone 40 mg daily for 4 days starting on the subsequent eighth and 16th days of the first, third and fifth courses of therapy.

With the first VAD patients were given allopurinol 300 mg daily for 2 weeks. Prophylaxis was routinely given against infection: cotrimoxazole 480 mg twice daily, increased to 960 mg twice daily after our first analysis had shown that 61% patients developed an infection (Anderson *et al.*, 1987), and ketoconazole 400 mg daily. Cimetidine 400 mg twice daily was used as prophylaxis for steroid-induced dyspepsia. All agents were given for 7 days every time patients commenced dexamethasone. In addition, patients with renal failure had an alkaline diuresis with the first course of VAD, together with dialysis if appropriate.

The assessment of response after six courses of VAD was according to the Chronic Leukaemia-Myeloma Task Force (1973), except that the definition of complete response was that described by Gore *et al.* (1989). The assessment of toxicity was according to standard criteria (Miller *et al.*, 1981).

### Results

From July 1984 to May 1992, 142 patients received VAD as remission induction therapy for myeloma. The patients' characteristics are shown in Table I. Seventy-five patients were previously untreated and 67 had either relapsed (31) or refractory disease (36). Of the relapsed patients, 29 had received prior alkylating agents and two had received doxorubicin. Of the refractory patients, 30 had received prior alkylating agents and six doxorubicin.

The median duration of follow-up (from first VAD to death, or to median date last seen in the surviving patients) is 37 months for untreated patients and 51 months for previously treated patients. The database was last updated in June 1993.

*Response*

Response to therapy is summarised in Table II. The median time to response was 6 weeks (range 5 days to 5 months), i.e. a median of two courses. Of the previously untreated patients, 20 (27%) achieved a complete response (CR) and 43 (57%) a partial response (PR). Of the previously treated patients, two (3%) achieved a CR and 39 (58%) a PR. The difference in CR rate between the two patient groups was statistically significant (Chi-square $P = 0.0003$). Seventeen of 31 (55%) patients with relapsed disease and 24/36 (66%) with refractory disease responded to VAD (chi-square $P = 0.46$).

*Subsequent therapy*

Previously untreated patients who did not have progressive myeloma after completion of VAD received melphalan 10 mg day$^{-1}$ for five days and prednisolone 50 mg day$^{-1}$ for 5 days every 6 weeks for a year. Previously treated patients were given 3 million units of alpha interferon thrice weekly for 1 year. In addition, 23 patients have undergone a bone marrow (BMT) or peripheral blood stem cell transplant (PBSCT).

*Bone marrow and peripheral blood stem cell transplant*

Since June 1986, 23 patients have received a transplant: eight allografts from matched sibling donors (four CR, three PR, one relapse), eight autografts (three CR, two PR, two stable plateau, one in relapse), and seven peripheral stem cell transplants following mobilisation with cyclophosphamide and granulocyte colony-stimulating factor (G-CSF) three CR, three PR, one stable plateau). The age ranges (years) were 35–48 for allogeneic, 25–49 for autologous and 37–59 for stem cell transplantation. All patients received 110 mg m$^{-2}$ melphalan and total body irradiation (1200 cGy in six fractions over 3 days) before bone marrow or stem cell infusion. PBSCT recipients also received alpha interferon maintenance therapy following reconstitution.

*Survival*

The median survival from starting VAD is 36 months for previously untreated patients and 10 months for the relapsed/refractory patients (Figure 1). For previously untreated patients the 75% survival was 14 months, and the 25% survival has not been reached. The median survival from diagnosis is 38 months for previously untreated patients and 39 months for patients with relapsed or refractory disease.

Analysis of survival by response after completion of VAD (with patients censored at the time of BMT), in previously untreated patients, has shown that the definition of CR used may be of prognostic importance as survival was longer in CR (not reached) than in PR patients (28 months; $P = 0.03$) (Figure 2).

The median duration of follow-up for those patients who received a transplant is 22 months from the date of first VAD. The median survival has not yet been reached for this subgroup of patients (Figure 3).

*Transplantation as consolidation therapy*

Of the 23 patients who had a transplant, eight have died. Two allograft recipients died of graft-versus-host disease at 1 month post transplant, and two died of infection at 2.5 and 3.5 months: one from fungal infection and one from combined cytomegalovirus infection and herpes simplex pneu-

**Table I** Patient characteristics

| | *Untreated* | *Relapsed and refractory* |
|---|---|---|
| Number | 75 | 67 |
| Male | 44 (59%) | 42 (63%) |
| Female | 31 (41%) | 25 (37%) |
| Median age (range) | 57 (25–80) | 59 (38–75) |
| Stage | 7 (9%) | 0 |
| IA | 19 (25%) | 12 (18%) |
| IIA | 1 (1.3%) | 1 (1.5%) |
| IIB | 25 (33%) | 42 (63%) |
| IIIA | 23 (31%) | 12 (18%) |
| IIIB | | |
| No courses median (range) | 6 (1–9) | 6 (1–15) |
| Median follow-up (months) from VAD | 37 | 51 |

**Table II** Response to therapy

| | *Untreated (75)* | *Relapsed or refractory (67)* |
|---|---|---|
| Complete response | 20 (27%) | 2 (3%) |
| Partial response | 43 (57%) | 39 (58%) |
| Stable | 7 (9%) | 15 (22%) |
| Progressed | 1 (1%) | 4 (6%) |
| Died | 4 (5%) | 7 (10%) |
| Median survival (months) | 36 | 10 |

**Figure 1** Survival for previously untreated patients and for those with relapsed refractory disease.

**Figure 2** Survival after VAD for patients according to response.

**Figure 3** Survival after a bone marrow or stem cell transplant.

monia. Four autograft patients died of progressive disease at 3.5–15.5 months post transplant. None of the seven patients who received PBSCT have died (6–14 months post transplant).

The median time for haematological reconstitution of neutrophils to $0.5 \times 10^9 l^{-1}$ was 19 days for alloBMT, 17 days for auto-BMT and 16 days for PBSCT patients. The median time to platelets $> 20 \times 10^9 l^{-1}$ was 21 days for allo-BMT, 23 days for auto-BMT and 13 days for PBSCT patients.

### *Toxicity of VAD*

Nine (6%) patients died within 30 days of the first VAD treatment. Their median age was 64 years (range 38–75 years). Five died of myeloma (8–24 days after VAD), three of infection and myeloma (4–30 days post VAD) and one of progressive multifocal leucoencephalopathy. A further patient discontinued chemotherapy after the first course and died of a cerebral infarction on day 51, and another died of pulmonary haemorrhage after the third course of VAD (day 62).

### *Symptomatic toxicity*

This is summarised in Table III. Seventy-six patients (54%) received antibiotics during VAD therapy: 31 (22%) needed oral antibiotics, 31 (22%) intravenous antibiotics and 14 (10%) received both intravenous and oral antibiotics. Chest infections were common, occurring in 40 (28%) patients. Documented bacteraemia occurred in 22 (14%) patients. Fifteen patients had Gram-positive isolates and seven (3%) Gram-negative isolates. Two patients died following bacteraemia, one of *Escherichia coli* bacteraemia on day 4 and the other of *Streptococcus pneumoniae* pneumonia and bacteraemia on the 31st day after VAD.

Despite ketoconazole prophylaxis, 18 (13%) patients developed oral candidiasis. Herpes zoster developed in five (4%) patients during VAD therapy.

Nausea and vomiting was mild – 30 (21%) patients reported vomiting (maximum WHO grade 2). Fifty-two (37%) patients developed dyspepsia. These patients were given continuous cimetidine prophylaxis. Constipation occurred in 42 (30%).

Steroid-associated oedema was seen in 38 (27%) patients, but did not necessitate a change of therapy. In seven patients (5%) mild heart failure was documented and treated with diuretics.

Problems with venous access occurred in 34 (24%) of 142 patients. The line fell out in 13 (9%), was replaced because of blockage in nine (6%), became infected and was removed in two (1%), became infected and was salvaged with antibiotics in three (2%) and in one (<1%) the Portacath insertion site became infected and required antibiotics. One line insertion was associated with a pneumothorax, and two (1%) patients developed a venous thrombosis (axillary and subclavian

**Table III** Symptomatic toxicity

| *Symptom* | *No. (%)* |
|---|---|
| Alopecia | 119 (84) |
| Antibiotics for infection | 76 (54) |
| Dyspepsia | 52 (37) |
| Constipation | 42 (30) |
| Paraesthesiae | 40 (28) |
| Oedema | 38 (27) |
| Line problems | 34 (24) |
| Nausea and vomiting | 30 (21) |
| Central nervous system | 19 (13) |
| *Candida* infection | 18 (13) |
| Heart failure | 7 (5) |

vein). In three patients with low platelet counts there was local bruising at the line insertion site.

### Discussion

VAD is an out-patient regimen with acceptable toxicity. Eighty-four per cent of previously untreated and 61% of patients with relapsed refractory myeloma responded to VAD. There was a significant difference in CR rate: 27% in previously untreated patients compared with 3% in patients with relapsed refractory disease ($P = 0.0003$). The median time to response was 6 weeks, i.e. after two courses of VAD. The median survival was 36 months for untreated patients and 10 months for those with relapsed refractory disease. For the untreated patients (with censoring at the time of BMT) survival is longer in patients who achieved a CR with VAD. Increased survival has been described after CR by other authors (Gore *et al.*, 1989; Samson *et al.*, 1989; Attal *et al.*, 1992). The importance of CR is currently being investigated in a number of randomised trials. High-dose therapy may improve the CR rate.

Selby *et al.* (1987) used high-dose melphalan (140 mg $m^{-2}$) to achieve a response rate of 78% (27%CR) and a median survival of 40 months in 41 previously untreated myeloma patients. However, toxicity was marked with 17% mortality in the first 2 months. In a MRC trial of combination therapy with ABCM (adriamycin, BCNU, cyclophosphamide and melphalan) *vs* melphalan alone, the response rates were similar [61% and 59% (8% CR)], however median survival was longer in the combination therapy arm: 32 *vs* 24 months (MacLennan *et al.*, 1992). Although comparing different patient populations, our previously untreated patients who received VAD then melphalan and prednisolone have a similar outcome to those reported by the above authors.

BMT and PBSCT are ways of increasing treatment intensity. We have found PBSCT to be well tolerated in patients up to 60 years old. PBSCT has a lower procedure-related mortality and morbidity in our hands than autologous or allogeneic BMT. These observations have been confirmed at our institution in 54 patients who have received PBSCT for leukaemia or lymphoma. There was a shorter duration of neutropenia, thrombocytopenia and hospitalisation than for historical controls receiving autologous BMT (Pettengell *et al.*, 1993).

The role of combination therapy in myeloma may be questioned after the meta-analysis of Gregory *et al.* (1992) showed melphalan and prednisolone to be superior for patients with a good prognosis and inferior for patients with a poor prognosis. This overview included 18 trials, of which 12 had been started in the 1970s. Caution should be exercised in extrapolating these results to modern chemotherapy with improved supportive care.

Patients with relapsed refractory disease had a higher response rate to VAD than that reported by Alexanian *et al.* (1986). Our patients generally had not received prior anthracyclines, and this may explain the differences. Our modification of VAD (omitting the steroids on days 9–12 and 17–20, and repeating courses every 21 days) has produced similar results to those initially described by Barlogie *et al.* (1984). However, some modifications of VAD may be detrimental: the NCI (Canada) gave a modified VAD regimen to patients with replapsed and refractory disease. Therapy was effectively reduced with a 2 h infusion VAD every 28 days. The observed response rate was only 27% with a median survival of 7.6 months (Browman *et al.*, 1992).

Methods to improve response in patients with relapsed/refractory disease are needed. High-dose therapy may not be the answer: high-dose melphalan produced a 66% response rate in patients with refractory disease, but the median survival was 10 months (Selby *et al.*, 1987). High-dose therapy with PBSCT produced a response in 4 of 11 patients with VAD-resistant myeloma (Ventura *et al.*, 1990). Allogeneic BMT may be an option for patients up to 55 years old – 49 patients with non-responsive or progressive myeloma treated with allo-BMT by the European Group for Bone Marrow Transplantation have a projected long-term survival of 30–40% (Gahrton *et al.*, 1991).

Interferon has shown activity in multiple myeloma. Randomised trials have been conducted using interferon after patients have responded to chemotherapy in myeloma. Some have shown an advantage for maintenance interferon (Mandelli *et al.*, 1990; Westin *et al.*, 1991; Cunningham *et al.*, 1993), however, others have not (Ludwig *et al.*, 1991). Randomised trials of interferon in combination with chemotherapy have shown higher response rates in the interferon group (Bjorkholm *et al.*, 1991; Ludwig *et al.*, 1991). Large, national studies are under way to address the optimal use of interferon.

New strategies are needed for improving the treatment of relapsed/refractory myeloma. Options include the use of maintenance interferon and dexamethasone (San Miguel *et al.*, 1990; Palumbo *et al.*, 1992), anti-interleukin 6 monoclonal antibodies (Klein *et al.*, 1990), or the use of agents to reduce resistance to chemotherapy (Jonsson *et al.*, 1992).

CR in multiple myeloma may be as important a prognostic factor for long-term survival as in other haematological malignancies. The high response rate of untreated myeloma to VAD encourages its continued use for remission induction therapy. Our present strategy in all VAD responders under 70 years of age is to consolidate with high-dose cyclophosphamide, harvest peripheral blood stem cells and proceed to melphalan and total body irradiation with PBSCT and maintenance interferon.

**Acknowledgements**
We are grateful for the support of colleagues in the NW region who have referred patients and the staff of the Day Ward, Ward 12 and Adult Leukaemia Unit at the Christie Hospital.

### References

ALEXANIAN R, BARLOGIE B AND DIXON D. (1986). High-dose glucocorticoid treatment of resistant multiple myeloma. *Ann. Intern. Med.*, **105**, 8–11.

ANDERSON H, SCARFFE JH, LAMBERT M, SMITH DB, CHAN CC, CHADWICK G, MACMAHON A, CHANG J, CROWTHER D AND SWINDELL R. (1987). VAD chemotherapy – toxicity and efficacy in patients with multiple myeloma and other malignancies. *Haematol. Oncol.*, **5**, 213–222.

ATTAL M, HUGUET F, SCHLAIFER D, PAYEN C, LAROCHE M, FOURNIE B, MAZIERES B, PRIS J AND LAURENT G. (1992). Intensive combined therapy for previously untreated aggressive myeloma. *Blood*, **79**, 1130–1136.

BARLOGIE B, SMITH L AND ALEXANIAN R. (1984). Effective treatment of advanced multiple myeloma refractory to alkylating agents. *N. Engl. J. Med.*, **310**, 1353–1356.

BJORKHOLM M FOR THE MYELOMA GROUP OF CENTRAL SWEDEN. (1991). Melphalan prednisolone versus melphalan prednisolone plus human alpha interferon therapy in patients with multiple myeloma, stages II and III. *Eur. J. Cancer*, **27** (Suppl. 4), s51–52.

BROWMAN GP, BELCH A, SKILLINGS J, WILSON K, BERGASEL D, JOHNSTON D AND PATER JL. (1992). Modified adriamycin-vincristine-dexamethasone (m-VAD) in primary refractory and relapsed plasma cell myeloma: an NCI (Canada) pilot study. *Br. J. Haematol.*, **82**, 555–559.

CHRONIC LEUKAEMIA-MYELOMA TASK FORCE. (1973). Proposed guidelines for protocol studies II. Plasma cell myeloma. *Cancer Chemother. Rep.*, **4**, 145–157.

CUNNINGHAM D, POWLES R, MALPAS JS, MILAN S, MELDRUM M, VINER C, MONTES A, HICKISH T, NICOLSON M, JOHNSON P, MANSI J, TRELEAVAN J, RAYMOND J AND GORE ME. (1993). A randomised trial of maintenance therapy with intron-A following high dose melphalan and ABMT in myeloma (abstract 1232). *ASCO*, **364**.

DURIE BGM AND SALMON SE. (1975). A clinical staging system for multiple myeloma. Correlation of measured myeloma cell mass with presenting clinical features, response to treatment, and survival. *Cancer*, **36**, 842–854.

GAHRTON G, TURA S, LJUNGMAN P, BELANGER C, BRANDT L, CAVO M, FACON T, GRANENA A, GORE M, GRATWOHL A, LOWENBERG B, NIKOSKELAINEN J, REIFFERS JJ, SAMSON D, VERDONCK L AND VOLIN L FOR THE EUROPEAN GROUP FOR BONE MARROW TRANSPLANTATION. (1991). Allogeneic bone marrow transplantation in multiple myeloma. *N. Engl. J. Med.*, **325**, 1267–1273.

GORE ME, VINER C, MELDRUM M, BELL J, MILAN S, ZUIABLE A, SLEVIN M, SELBY PJ, CLARKE PI, MILLAR B, MAITLAND JA, JUDSON IR, TILLYER C AND MALPAS JS. (1989). Intensive treatment of multiple myeloma and criteria for complete remission. *Lancet*, **ii**, 879–885.

GREGORY WM, RICHARDS MA AND MALPAS JS. (1992). Combination chemotherapy versus melphalan and prednisolone in the treatment of multiple myeloma: an overview of published trials. *J. Clin. Oncol.*, **10 (2)**, 334–342.

JONSSON B, NILSSON K, NYGREN P AND LARSSON R. (1992). SDZ-PSC-833 – a novel potent in vitro chemosensitiser in multiple myeloma. *Anticancer Drugs*, **3**, 641–646.

KLEIN B, ZHANG XG, JOURDAN M, BOIRON JM, PORTIER M, LU ZY, WIJDENES J, BROCHIER J AND BATAILLE R. (1990). Interleukin 6 is the central tumor growth factor *in vitro* and *in vivo* in multiple myeloma. *Eur. Cytokine Netw.*, **1**, 193–201.

LUDWIG H, COHEN AM, HUBER H, NACHBAUR D, JUNGI WF, SENN H, GUCZLER P, SCHULLER J, ECKHARDT S, SEEWANN HL, CAVALLI F, FRITZ E AND MICKSCHE M. (1991). Interferon alfa-2b with VMCP compared with VMCP alone for induction and interferon alfa-2b compared to controls for remission maintenance in multiple myeloma; interim results. *Eur. J. Cancer*, **27** (Suppl. 4), s40–45.

MACLENNAN ICM, CHAPMAN C, DUNN J AND KELLY K. (1992). Combined chemotherapy with ABCM versus melphalan for treatment of myelomatosis. *Lancet*, **339**, 200–205.

MANDELLI F, AVVISATI G, AMADORI S, BOCCADORO M, GERNONE A, LAUTO VM, MARMONT F, PETRUCCI MT, TRIBALTO M, VEGNA ML, DAMMACCO F AND PILERI A. (1990). Maintenance treatment with recombinant interferon alfa-2b in patients with multiple myeloma responding to conventional induction chemotherapy. *N. Engl. J. Med.*, **322**, 1430–1434.

MILLER AB, HOOGSTRATEN B AND STAQUET M. (1981). Reporting results of cancer treatment. *Cancer*, **47**, 207–214.

PALUMBO A, BOCCADORO M, GARINO LA, GALLONE G, FRIERI R AND PILERI A. (1992). Multiple myeloma: intensified maintenance therapy with recombinant interferon alpha-2b plus gluco-corticosteroids. *Eur. J. Haematol.*, **49**, 93–97.

PETTENGELL R, MORGENSTERN GR, WOLL PJ, CHANG J, ROWLANDS M, YOUNG R, RADFORD JA, SCARFFE JH, TESTA NG AND CROWTHER D. (1993). Peripheral blood progenitor cell transplantation in lymphoma and leukaemia using a single apheresis. *Blood*, **82**, 3770–3777.

SAMSON D, NEWLAND A, KEARNEY J, JOYNER M, MITCHELL T, BARRETT AJ, GAMINARA E, VAN DE PETTE J, MCCARTHY D, ASTON A, HAMON M AND EVANS M. (1989). Infusion of vincristine and doxorubicin with oral dexamethasone as first-line therapy for multiple myeloma. *Lancet*, **ii**, 882–885.

SAN MIGUEL JF, MORO M, BLADE J, GUERRAS L, HERNANDEZ J, JIMENNEZ GALINDO R, ORTEGA F AND GONZALEZ M. (1990). Combination of interferon and dexamethasone in refractory multiple myeloma. *Haematol. Oncol.*, **8**, 185–189.

SELBY PJ, MCELWAIN TJ, NANDI AC, PERRIN TJ, POWLES R, TILLYER CR, OSBOURNE RJ, SLEVIN ML AND MALPAS JS. (1987). Multiple myeloma treated with high dose intravenous melphalan. *Br. J. Haematol.*, **66**, 55–62.

VENTURA GJ, BARLOGIE B, HESTER JP, YAU JC, LEMAISTRE CF, WALLERSTEIN RO, SPINOLO JA, DICKE KA, HOROWITZ LH AND ALEXANIAN R. (1990). High dose cyclosphosphamide, BCNU and VP16 with autologous blood stem cell support for refractory multiple myeloma. *Bone Marrow Transplant.* **5**, 265–268.

WESTIN J, CORTELEZZI A, HJORTH M, RODJER S, TURESSON I AND ZADOR G. (1991). Interferon therapy during the plateau phase of multiple myeloma: an update of the Swedish Study. *Eur. J. Cancer*, **27** (Suppl. 4), 45–48.

WOODRUFF R. (1981). Treatment of multiple myeloma. *Cancer Treat. Rev.*, **8**, 225–270.